# Comparing closure compliance and ease of use for consumer product packs designed to reduce access to children

**DOI:** 10.1371/journal.pone.0284346

**Published:** 2024-04-22

**Authors:** Annalise Richmond, David C. Schwebel, Clara Ng Pak Leung, Elke Vallez, Heidi Lesage, Raf Degeyter, Zhiwu Liang, Gerard Stijntjes

**Affiliations:** 1 Procter and Gamble, Strombeek Bever, Belgium; 2 Department of Psychology, University of Alabama at Birmingham, Birmingham, Alabama, United States of America; Wroclaw University of Environmental and Life Sciences: Uniwersytet Przyrodniczy we Wroclawiu, POLAND

## Abstract

Child impeding packs are difficult for children to open so protect them from unintended access to hazardous contents inside the pack. However, if packs are difficult for adults to open, in normal usage scenarios, this may result in a higher occurrence of packs being left open. This research explores differences in ease of usage and closure compliance between two types of child impeding packs of liquid laundry detergent capsules. The two packs, “Pinch & Lift” and “Press & Lift”, had different opening and closing mechanisms. “Press & Lift” also included an audible “click” signal to confirm complete closure to the user. The research was performed across two studies. In both studies, the packs were used in participants’ homes according to their usual storage and usage practices as replacements to their current liquid laundry detergent capsule pack. All participants had small children living with them in their household. In Study 1, self-reported closure and ease of use data was collected from 99 adult participants in Germany. They used each package in their home for 10 days. Study 2 extended Study 1 by measuring closure rates with an objective assessment using in pack sensors for a 10-day period for each pack. Self-reported closure and ease of use data were also collected. Study 2 was conducted with 87 participants in the United Kingdom. Results across both studies showed “Press & Lift” with the audible “Click” close signal to be rated by participants to be significantly easier to open and close and have a higher self-reported closure rate than “Pinch & Lift”. In addition, Study 2 results demonstrated higher closure rates using “Press & Lift” based on the sensor-measured closure compliance. Together, the results suggest transition to a pack with a mechanism that is child impeding and easier for an adult to use with an audible closure signal, like the “Press & Lift” system, has potential to reduce child access to a capsule from a pack by reducing the likelihood of the pack being left open by the adult user. Ultimately, such packs could protect children from potential poisoning injury across a range of consumer products.

## Introduction

Risk of child poisoning occurs when young children access hazardous products in containers that are not closed properly by adult users. For example, young children have experienced unintentional poisoning incidents through exposure to Liquid Laundry Capsules because they access laundry packs that are not closed securely [1]. In response to such incidents, plus extensive empirical data indicating the effectiveness of strategies like child-impeding closures [2] and opaque packaging [3], legislative bodies in Europe have protected consumer interest by requiring industry to sell liquid laundry capsules in opaque packaging and to inform consumers through on pack labeling about the hazards present and the need for safe storage.

Ultimately, however, safe use and storage of potentially dangerous household products such as liquid laundry capsules rely not just on safety engineering by industry but also on decisions and behaviors by users [4, 5]. Will they consistently and reliably close containers to keep children safe? Previous research suggests some users either intentionally or unintendedly leave child impeding closures open and store packs in places accessible to young children [1]. This behavior may contribute to unintentional pediatric exposures [1, 6].

The present research was designed to test whether an alternative type of packaging might increase compliance with proper and consistent closure of packaging by adult users. A large body of research suggests health-related human behavior and habits are resistant to change [7]. Thus, the ideal solution is to identify a package which is quick, easy and convenient for adults to open and close, but which simultaneously meets standards for child-impeding packaging to reduce the risk of children handling the hazardous product, with the possible result of an accidental exposure leading to poisoning. The packaging also must be resistant to forced entries by children.

From a health behavior theory perspective, a package that is quick, easy and convenient to open and close should increase the user’s self-efficacy to accomplish the task of opening and closing the package for safety. Self-efficacy is widely cited as a critical component of many leading health behavior change theories, including the Health Belief Model [8] and Protection Motivation Theory [9]. When someone feels able and confident to complete a task efficiently, they are more likely to engage in that task [10, 11]. With other theoretical mechanisms for behavior change in place, a quick and easy closure mechanism should improve compliance with health-promoting behaviors such as closing packs containing hazardous materials to reduce risk of unintentional exposure to children.

This research considers therefore the effectiveness of a newly designed package to store products like liquid laundry capsules in a manner that encourages users to open and re-close the package properly and repeatedly. Two studies were conducted with caregivers of young children to evaluate the packages. The first evaluated whether the new package yielded higher rates of self-reported closure and ease of use compared to a current, commercially available liquid laundry capsule package normally used in the participants’ home. The second extended the first study by considering not just self-reported closure and ease of use but also objectively measuring actual closure rates using sensors hidden inside the package. Given our belief that it was easier to open and close, we hypothesized the new package would result in higher rates of closure and higher ratings of ease of use.

## Study 1

### Materials and methods

#### Overview of methods

The first study, conducted in Germany, comprised of a two-armed randomized trial to investigate self-reported closure of the two laundry liquid capsule packages during normal in-home usage. We also investigated self-reported ease of use and collected qualitative data at the end of the research concerning respondents’ thoughts about the two types of packages they had used.

All participants provided written informed consent. Study protocols were approved by the Ethics Committee in Human Research of the University of Leuven, Belgium (Reference Number G-2021 04 2052).

#### Participants

Recruitment and fieldwork were managed by the market research agency Plan Marktforschung GMBH. 99 adults living in Germany (mean age = 35 years, SD = 7.2, range = 20–57; 16% male, 84% female) participated. They were recruited to participate in consumer research (with no explicit mention of the safety focus) from the agency’s database of adults interested in such research. One additional participant started the study but did not finish and was omitted from all analyses.

Inclusion criteria were adults aged 18–65 years who took primary responsibility for the laundry in their household, washing at least 5 loads per week and thus providing sufficient opening and closing behavior for us to collect data for analysis. Participants were required to have at least one child aged less than 5 years living with them in the household. In addition, participants were required to have used Ariel Liquid Laundry Capsules sold in tubs in the previous 3 months as their usual detergent, as this product formed the control product in the experiment. Exclusion criteria were minimal and included only physical limitations that precluded valid participation in the research, such as vision or upper limb extremity disabilities that prevented them from independently seeing, opening and closing packages.

Sample size was driven by the need to distinguish statistical differences in closure compliance rates between the two test packs. Because inclusion criteria required completion of at least 5 loads of laundry per week, it was assumed that within the 10-day usage period at least 8 loads of laundry would be completed, each resulting in occasions when discrete opening and closing would be best to keep children in the household safe. Based on qualitative pilot research, consumers were expected to close the pack at least 70% of times after they opened the pack. With this assumption, a minimum sample size of 80 respondents was required to detect a 7% difference between the packaging closure rates with 80% power and 95% confidence level.

#### Materials

**Fig 1:**
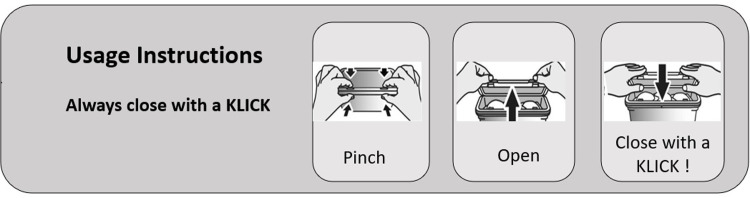
Pack “Pinch & Lift” opening and closing mechanism.

All packs were filled with 35 Ariel Liquid Laundry Capsules, which were commercially available in Europe at the time of the research. The “Pinch & Lift” pack used current commercially available packaging. It had a hinged lid with two front latches that opened with a simultaneous “pinch” (using fingers and thumbs from both hands) and “lift” movement. To close, both latches are clicked back into place to signal full re-engagement as the lid is pressed down into closed position. (Fig 1).

**Fig 2:**
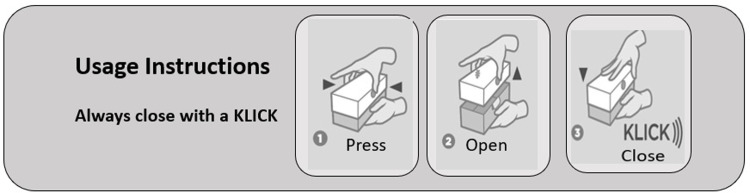
Pack “Press & Lift” opening and closing mechanism.

The 2^nd^ Pack (Press & Lift) was developed with a modified opening and closure mechanism. It had an independent lid with front and back buttons. To open, the pack is held in one hand and the finger and thumb from the second hand are used to simultaneously “press” the two buttons and “lift” the lid. To close, both buttons “click” back into place and signal full re-engagement as the lid is pressed back down into position. (Fig 2).

Both packs were certified as being child impeding by an industry approved independent testing center (AIJU, Alicante, Spain). They were both labeled according to market practices with Ariel branding, capsule dosage guidance, customized pack opening and closing usage instructions, capsule formulation information, and industry-standard detergent hazard warning labels.

#### Procedure

Following consent processes, each respondent was sent a preliminary questionnaire to gather information about their satisfaction with their current detergent and their habits and practices when doing laundry. Next, a test pack was mailed to each participant. They were instructed to place their current laundry detergent in a different location and replace it with the test pack, storing and using the test pack according to their normal laundry habits and practices. After the 10-day usage period, participants returned the test pack by mail using a pre-paid envelope. They then completed an online questionnaire to assess usage experience with the test pack.

Upon receipt of the first pack in the mail, the research team mailed the second pack to each participant and the procedure was repeated. Participants replaced their current detergent with the second test pack, used it for 10 days, and then returned it by mail. A second online questionnaire was completed concerning experience with the second pack.

Order of pack usage was randomised across participants, with half using “Pinch & Lift” first and the other half using “Press & Lift” first.

#### Outcome measures

We considered three primary outcomes: storage location, self-reported closure habits, and usage experience ratings. They were assessed through questions with fixed responses.

Storage was assessed via the question, “Can you tell me about the location within your home that best describes where you store the laundry detergent?”, which was answered by checking one of four fixed responses: “Kitchen”, “Bathroom”, “Utility room/Laundry room”, or “Other”.

Closure habits were assessed via the question, “When you finished the laundry for the day, how often did you close the pack?”, which was answered using a 5-point scale ranging from “Always” to “Never”. In addition, the question “When you did multiple loads of laundry during the day, how often did you close the pack in between loads?” was asked. This was answered using the same 5-point scale.

Usage experience acceptance ratings were asked via a series of questions, “How would you rate the packaging of the laundry detergent for the following attributes–(a) Easy to open, (b) Easy to close, (c) Being child safe, and (d) Simplifying my laundry. Each question was answered by checking a fixed response using a 5-point scale (1 to 5) ranging from “Poor” to “Excellent”. To ease interpretation of results for user experience, we assigned a 100-point scale, computed as ((5-point scale response– 1) * 25) to yield a possible range of 0 to 100 for user experience ratings, with higher scores reflecting higher ratings.

Qualitative insights about the respondent’s usage experience were collected using the following question, “Please write a short review about your experience–like an online Amazon review. Please try to capture all of your product and packaging related experiences–both positive and negative”.

#### Statistical analyses

Following consideration of descriptive data, we examined differences in self-reported closure of the pack by computing mixed models using the Glimmix procedure in SAS 9.4 (100 SAS Campus Drive, Cary, NC 27513–2414, USA). For both outcome variables–closure after finishing laundry for the day and closure between loads when completing multiple loads of laundry during the day–participant’s responses served as the response variable; product, order and the product*order interaction as fixed effects; and participant as a random effect.

Next, a Mixed ANOVA model in JMP 16.1 (100 SAS Campus Drive, Cary, NC 27513–2414, USA) was applied to the four usage experience ratings, allowing us to compare the mean difference in ratings between two products (“Pinch & Lift” and “Press & Lift”). Product, order and the product*order interaction were entered as fixed effects and participant as a random effect. The primary outcome was least squares mean for each product.

Finally, we considered qualitative responses concerning usage experience.

### Results

The majority of participants stored their laundry capsules either in the bathroom (n = 36; 36%) or a utility/laundry room (n = 33; 33%), followed by the kitchen (n = 18; 18%) and other locations (n = 12; 12%).

Table 1 shows data concerning participants’ reports about closing the pack at the end of the day. As shown, 67% of participants (n = 66) stated they always closed the “Pinch & Lift” pack and 82% stated they always closed the “Press & Lift” pack. This difference was statistically significant (*p* = 0.004). For both packs, 4% of participants said they never closed it (*p* = 0.982).

**Table 1:** Closure of pack during the research. Percentage (and Standard Error) of participants who reported re-closing pack at the end of the day.

	Pinch & Lift n; P (SE%)	Press & Lift n; P (SE%)	Effect Size h
% Always closed	66; 67% (4.8%)	81; 82% (3.9%)**	.34
% Mostly closed	12; 12% (3.3%)	10; 10% (3.0%)	.07
% Sometimes closed, sometimes not	8; 8% (2.7%)	1; 1% (1%)*	.38
% Mostly don’t close	8; 8% (2.7%)	3; 3% (1.7%)	.23
% Never closed	5; 4% (2.2%)	4; 4% (2%)	.002

* *p* < .05. ** *p* < .01.

Just 5% of participants using “Pinch & Lift” and 3% using “Press & Lift” stated they did not complete multiple laundry loads in the same day. Among the remainder of the participants, as shown in Table 2, 63% stated they closed the “Pinch & Lift” pack between laundry loads on the same day most of the time and 81% stated this about the “Press & Lift” pack. This difference was statistically significant (*p*<0.0001). There also was a statistically significant difference among those reporting they never closed the packs between laundry loads, with 14% of participants using Pinch & Lift and 7% using “Press & Lift” stating this (*p* = .0331).

**Table 2:** Closure of pack during the research. Percentage (and Standard Error) of participants who reported re-closing pack in between different loads.

	Pinch & Lift n; P (SE%)	Press & Lift n; P (SE%)	Effect Size h
% Always closed	0% (0%)	0% (0%)	-
% Mostly closed	62; 63% (4.9%)	80; 81% (4%)**	.41
% Sometimes closed, sometimes not	14; 14% (3.5%)	8; 8% (2.7%)	.18
% Mostly don’t close	2; 2% (1.4%)	1; 1% (1.0%)	.08
% Never closed	16; 14% (3.8%)	7; 7% (2.6%)*	.24

*Note*. 5 individuals in Pinch & Lift and 3 individuals in Press & Lift condition did not complete multiple back to back laundry loads, so data are missing for those conditions.

* *p* < .05. ** *p* < .01.

Table 3 lists usage experience ratings from the participants for both packs. Participants reported the “Press & Lift” pack was easier to open (mean difference of 87 vs 55 on the transformed 100-point scale, *p* < .0001), easier to close (88 vs 72, *p* < .0001), and simplified their laundry (83 vs 71, *p* = .00016). There was no significant difference in ratings on being child safe (82 vs 83, *p* = .56).

**Table 3:** Least squares mean (and standard error) of usage experience ratings for both packs from mixed model.

	Pinch & Lift Mean (SE)	Press & Lift Mean (SE)	Cohen’s d	df	Fixed Effect Pack *F*	Fixed Effect Order *F*	Fixed Interaction Effect *F*	Random Effect: REML estimation (SE)
Easy to open	55(2.8)	87(2.8)**	1.14	1,194	66.61**	2.71	2.12	0 (0)
Easy to close	72(2.3)	88(2.3)**	0.73	1,97	26.04**	2.11	3.50	41(54)
Being child safe	83(2.2)	82(2.2)	0.05	1,97	0.34	4.42*	0.38	66(50)
Simplifying my laundry	71(2.3)	83(2.3)**	0.55	1,97	15.40**	6.64*	2.94	61(55)

* *p* < .05. ** *p* < .01.

Table 4 lists selected comments collected from participants in the qualitative reviews to help explain differences in the quantitative survey data concerning the negative impressions about usability of the “Pinch & Lift” pack and more positive feelings about the “Press & Lift” pack’s usability and safety.

**Table 4:** Selected qualitative statements about the two packaging systems.

Participant reports about the “Pinch & Lift” pack	Participant reports about the “Press & Lift” pack
✔ “I can only rate the packaging negatively. The packaging is made of plastic and the child lock is very complicated to open even for adults.” ✔ “Unfortunately, the pack is awkward to open because the plastic clips are very hooked.” ✔ “Packaging is way too difficult to open!” ✔ “I do not like the packaging because it is very stupid to open.” ✔ “Packaging hard to open which makes it child safe but also hard for parents to open.”	✔ “The packaging is easy and intuitive to open for adults and the parental control works well.” ✔ “With the box, FINALLY the packaging has been revised: easy to handle.” ✔ “Great new packaging can be opened easily, yet with parental control. I am really excited!” ✔ “The closure is simple but totally ingenious!” “The box is top: 100 x better than the old tub. I would have loved to send you my box back and keep the new one.”

### Discussion

Study 1 suggests participants found the “Press & Lift” pack easier to open and close than the “Pinch & Lift” pack but equally safe. They also reported closing it more consistently. The primary limitation of Study 1 was that it relied entirely on self-report data. Study 2 was designed to replicate Study 1 in a different country and culture, and to incorporate objective measurement of pack closure as well as collecting self-reported data from participants.

## Study 2

### Materials and methods

#### Overview of methods

Like Study 1, Study 2 was a two-armed trial. It was conducted in the United Kingdom (UK), a country with a different culture and slightly different laundry practices than Germany (in particular, laundry facilities in UK homes tend to be in kitchens whereas those in Germany are more frequently in bathrooms or utility/laundry rooms). Study 2 also incorporated two key methodological changes. First, objective measures of actual user closure were collected through devices disguised in the packs as well as through self-reported behaviour concerning pack closure. Second, given the lack of order effects in Study 1 we used an A-B-A research design in Study 2 to examine potential effects on participants having to stop using their normal packaging. Specifically, we evaluated closure habits across two phases (“Pinch & Lift” and then “Press & Lift”, non-randomized) but usage experience across three phases (with the familiar “Pinch & Lift” package, the novel “Press & Lift” package, and then again with the “Pinch & Lift” package to determine whether effects based on order of condition presentation emerged).

#### Participants

Recruitment and execution of the research was managed by Launchpad Research Limited. 87 adults (mean age = 33 years, SD = 8, range = 18–61; 5% male, 95% female) participated. One additional participant started the study but did not finish and was omitted from all analyses. As in Study 1, participants were recruited to participate in consumer research (no explicit mention of the safety focus) from a database of adults interested in such research in the city area of Newcastle Upon Tyne, United Kingdom. Inclusion criteria, exclusion criteria, and sample size calculations were identical to Study 1.

All participants provided written informed consent. Study protocols were approved by the Ethics Committee in Human Research of the University of Leuven, Belgium (Reference Number G-2021 04 2052).

#### Materials

The same types of packs were used in Study 2 as Study 1, but both types were modified to be fitted with hidden battery-powered sensors that digitally monitored opening and closing of the child impeding lid using magnetic proximity detection sensors. The sensors, including the electronic board, battery, wiring and contacts, were fixed and integrated into the packs in separate cavities located at the bottom of the pack for the “Pinch & Lift” pack and inside the lid for the “Press & Lift” pack. They were fully concealed and protected from the user and did not interfere with usage mechanism of the packs.

#### Procedure

Study 2 followed the same procedure as Study 1, with the revision that order was not randomised. Instead, all participants used the “Pinch & Lift” pack first, followed by the “Press & Lift” pack, and then a second trial with the “Pinch & Lift” pack. Order was not a meaningful factor in Study 1 and this A-B-A research design allowed us to evaluate for any possible effect of user impressions about the new “Pinch & Lift” design across two usages of the more familiar “Press & Lift” system. After each usage, the packs were returned by mail, dismantled and data recorded by the sensors were extracted for analysis. All other data were collected through online survey, as in Study 1.

#### Outcome measures

We considered four primary outcomes: storage location, self-reported opening and closure, usage experience, and sensor-recorded opening and closure. Storage location, self- reported opening and closure, and usage experience were assessed through the same fixed response questions and response scales used in Study 1.

The sensors recorded opening and closure behaviour continuously over the 10-day study periods. For each participant we considered three measures: (a) the total number of opening interactions the participant had with the pack over the 10 days, (b) the total number of safe reclosures (which we defined as reclosing within 1 minute), and (c) the percentage of safe re-closures. We also examined the percentage of total time that each pack was closed over the 10-day period, recorded on a five-point scale (>99%, 75–98%, 50–74%, 25–49%, <24%).

#### Statistical analyses

Following consideration of descriptive data and replicating the statistical analyses from Study 1, we first computed mixed models using the Glimmix procedure in SAS 9.4 (100 SAS Campus Drive, Cary, NC 27513–2414, USA) to evaluate participant’s self-reported reclosure at the end of the day and between multiple loads of laundry during the same day.

Next, we considered closure data measured objectively using the sensors. Mixed ANOVA models were computed in JMP 16.1 (100 SAS Campus Drive, Cary, NC 27513–2414, USA) and mean differences were computed between the two packs on whether the pack was closed within a minute of being opened, with pack as a fixed effect and participant as a random effect. A mixed model using the Glimmix procedure was used to assess the percentage of time the sensor was closed, as measured with the sensor, with the five categories of closure serving as the response variable, pack as a fixed effect, and participants as a random effect.

Finally, we computed mixed models using the Glimmix procedure to evaluate usage experience, replicating the analyses used in Study 1 with participant ratings as the response variable, pack as a fixed effect, and participant as a random effect.

### Results

Different from Study 1 patterns in Germany, among this UK sample in Study 2, the majority of participants stored their laundry capsules either in the kitchen (n = 65; 75%) or a utility/laundry room (n = 20; 23%). As expected, self-reported closure of packs was statistically associated with sensor-reported closure, Spearman’s ρ = 0.34, *p*<0.0001.

As shown in Table 5, significantly more participants reported always closing the pack when returning the “Press & Lift” pack at the end of the day (80%) compared to when using the “Pinch & Lift” (47%; *p <* .0001). Significantly more participants reported only sometimes closing the pack (rather than mostly or always closing it) when returning the “Pinch & Lift” pack at the end of day (22%) compared to when using “Press & Lift” pack (5%; *p =* .0002).

**Table 5:** Self-reported closure of pack during the research. Percentage (and Standard error) of participants who reported re-closing pack at the end of the day.

	Pinch & Lift n; P (SE%)	Press & Lift n; P (SE%)	Effect Size h
% Always closed	41; 47% (5%)	70; 80% (4%)**	.71
% Mostly closed	10; 11% (3.4%)	5; 6% (2.5%)	.21
% Sometimes closed, sometimes not	19; 22% (4.4%)	4; 5% (2.2%)**	.54
% Mostly don’t close	8; 9% (3.1%)	3; 3% (2.0%)	.24
% Never closed	8; 9% (3%)	4; 5% (2%)	.18

*Note*. 1 individual in the Pinch & Lift condition responded that they transferred the product into different packaging which they usually did not close. 1 individual in the Press & Lift condition responded that they transferred the product into different packaging which they usually closed.

* *p* < .05. ** *p* < .01.

As shown in Table 6, when using the “Press & Lift” pack, significantly more participants reported always closing the “Press & Lift” pack between wash loads during the day (75%) compared to when using the “Pinch & Lift” pack (41%; *p* < .0001). Correspondingly, just 9% of participants stated they sometimes closed the “Press & Lift” pack between loads whereas 25% sometimes closed the “Pinch & Lift” pack (*p* = 0.0058). There also was a non-significant trend for participants to never close the “Pinch & Lift” pack (13% of participants) more often than the “Press & Lift” pack (6%) between wash loads during the day (*p* = .0526).

**Table 6:** Self-reported closure of pack during the research. Percentage (and Standard Error) of participants who reported re-closing pack in between different loads.

	Pinch & Lift n; P (SE%)	Press & Lift n; P (SE%)	Effect Size h
% Always closed	36; 41% (5%)	65; 75% (5%)**	.69
% Mostly closed	11; 13% (3.6%)	6; 7% (2.7%)	.20
% Sometimes closed, sometimes not	22; 25% (4.7%)	8; 9% (3.1%)**	.44
% Mostly don’t close	3; 3% (2.0%)	2; 2% (1.6%)	.07
% Never closed	11; 13% (4%)	5; 6% (2%)	.24

*Note*. In the Pinch & Lift condition, 1 individual did not do multiple washes on one day, 1 individual never closed the packaging between washes because the closure system was broken, 1 individual transferred the product into different packaging which they usually did not close in between washes, and 1 individual transferred the product into different packaging which they usually did close between washes. In the Press & Lift condition, 1 individual transferred the product into different packaging which they usually did not close in between washes.

* *p* < .05. ** *p* < .01.

As shown in Table 7, the “Press & Lift” pack sensors recorded a significantly higher number (7) and percentage (82%) of reclosures in less than 1 min compared to the “Pinch & Lift” pack sensors (4 and 51%, respectively; both *p* < .0001). There also were somewhat more interactions and closings with the “Press & Lift” pack than the “Pinch & Lift” pack (*p* = 0.011).

**Table 7:** Sensor-reported closure of pack during the research. Least Squares Mean of Number (percent) of pack interactions that were re-closed in less than 1 min from mixed model.

	Pinch & Lift N or P (SE)	Press & Lift N or P (SE)	Cohen’s d / Effect Size h	df	Fixed Effect Pack *F*	Random Effect: REML estimation (SE)
No. interactions (mean)	8 (0.6)	10 (0.7)*	0.45	1,69.72	6.82*	19 (5)**
No. reclosures < 1 min (mean)	4 (0.6)	7 (0.6)**	0.68	1,84.71	19.51**	10 (4)**
% reclosures within 1 min	51% (3.3%)	82% (3.4%)**	1.15	1,76.1	51.14**	130.8 (108.1)

No. = Number. * *p* < .05. ** *p* < .01.

As shown in Table 8, the “Press & Lift” pack sensors recorded significantly more participants closing the pack within 1 minute after opening than “Pinch & Lift”, with significant differences at the 100% of the time (41% vs 15%, *p*<0.0001) and 75–99% of the time (36% vs 16%, *p* = 0.004) levels as well as the 25–49% of the time (4% vs 28%, *p*<0.0001) and 0–24% of the time levels (4% vs 20%, *p =* 0.0006).

**Table 8:** Proportion of usages where sensors confirmed pack to be reclosed within 1 minute after opening, based on the actual number of interactions during the total 10-day usage period. Percent (and Standard Error) of participants with reported closure.

	Pinch & Lift n; P (SE%)	Press & Lift n; P (SE%)	Effect Size h
100% of time	12; 15% (4%)	31; 41% (5.7%)**	0.61
75–99% of time	13; 16% (4.1%)	27; 36% (5.5%)**	0.46
50–74% of time	17; 21% (4.6%)	11; 15% (4.1%)	0.17
25–49% of time	22; 28% (5%)	3; 4% (2.2%)**	0.71
0–24% of time	16; 20% (4.5%)	3; 4% (2.3%)**	0.54

* *p* < .05. ** *p* < .01.

Table 9 shows data from the evaluation of user experience across all three time points, including the final phase with a second exposure to “Pinch & Lift”. As shown and consistent with Study 1, the “Press & Lift” pack was rated significantly higher than the “Pinch & Lift” pack for both ease of opening and closing as well as for simplifying the laundry process. This was particularly true when comparing the “Press & Lift” ratings to the second “Pinch & Lift” ratings, after both packs had been used and experienced. Consistent with study 1, both packs were scored about equally by users for keeping children safe.

**Table 9:** Usage experience assessment across three phases of study. Least Squares Mean (and Standard error) of usage experience ratings for both packs from mixed model.

	Pinch & Lift Baseline Mean (SE)	Press & Lift Test Phase Mean (SE)	Pinch & Lift Second Exposure Mean (SE)	Cohen’s d: Baseline v. Test; Baseline v. 2nd Exposure; Test v. 2nd Exposure	df	Fixed Effect Pack *F*	Random Effect: REML estimation (SE)
Easy to open	69 (3.2)^a^	73 (3.2)^b^	58 (3.2)^a^^b^	0.13; 0.37; 0.50	2,258	5.95**	0 (0)
Easy to close	87 (2.2)^c^	87 (2.2)^d^	75 (2.2)^c^^d^	0.02; 0.55; 0.60	2,172	10.19**	32 (28)
Being child safe	85 (2.6)	86 (2.6)	86 (2.6)	0.04; 0.00; 0.04	2,258	0.04	0 (0)
Simplifying my laundry	83 (2.8)^e^	79 (2.8)^f^	67 (2.8)^e^^f^	0.18; 0.46; 0.62	2,172	0.25**	0.45 (42)

^a^*p* = 0.0396. ^b^*p* = 0.0029 ^c^*p* = 0.0003. ^d^*p* = 0.0005 ^e^*p* = 0.0001. ^f^*p* = 0.0087

### Discussion

Results from Study 2 concord with those from Study 1. Participants reported a consistent preference for the “Press & Lift” pack over the “Pinch & Lift” pack. They also reported closing it more frequently, data that were confirmed through the objective data collected by disguised sensors. Together, the findings imply child poisoning risk might be reduced through use of the “Press & Lift” pack instead of the “Pinch & Lift” one.

Results in Study 2 also extend the findings to a new culture and setting. Of particular importance, homes in the UK tend to have laundry facilities in the kitchen, a location where children may be more likely to be present, rather than in laundry/utility rooms or bathrooms.

## General discussion

Together, the two studies suggest the “Press and Lift” packaging is preferred by users, leads to higher rates of closure during use, and is rated as easier to open and close. A recent study amongst liquid laundry capsule consumers [1] showed that people who have had safety incidents with liquid laundry capsules state that their child gained access to the capsule about equally often in one of three ways: 1) when the capsule was left out of the packaging on a reachable surface, 2) by accessing the capsule from a pack that had been left open, and 3) by accessing a capsule from a pack that has been closed. Transition to a pack like the “Press & Lift” system has potential to reduce child poisoning incidents that are a result of the second cause, when children access the capsule from a pack that was left open by an adult user.

Since 2018 all laundry packs manufacturers were invited to adopt an industry wide agreement (PSP 3) to redesign their laundry capsule packaging to meet the Child Impeding Closure (CIC) certification and reduce the risk of unattended young children opening closed packs. Such steps have likely been successful in making packs harder for children to open, but they also have become more difficult and time consuming for adult users to open as well. This may have led many users to leave packs open, as many individuals appreciate ease and convenience in accomplishing daily tasks like laundry and may not bother to close packs if they are difficult to open and close quickly, conveniently, and efficiently. In other words, Child Impeding Closures that are difficult to open may actually lead to an increase in packs that are left open, limiting the intended impact of the CIC mechanism. From a theoretical perspective, use of packages like “Press & Lift” that can be easily and quickly opened and closed is likely to increase self-efficacy among users to accomplish health-related behaviour change. If a task is easy to accomplish, users will gain confidence to take that action and reduce risks of a negative health outcome, such as child exposure to hazardous products [8–11].

At the heart of our findings, therefore, is the fact that participants rated “Press & Lift” as easier to use. From a behavioural science theory perspective, although we did not directly measure the construct our findings suggest we may have altered user’s self-efficacy to accomplish the task of closing the packaging after use. Multiple leading health behaviour theories cite the need or advantage of having self-efficacy for an individual to change their health-related behaviour patterns [8–12]. If someone feels they are capable of engaging in a health-promoting task like changing their diet or stopping cigarette-smoking, they are more likely to do so. In a recent study of a large sample of adolescents, for example, those who felt higher self-efficacy to restrict sugar-sweetened beverages intake were best able to restrict their intake [13]. Packs with design features like the “Press & Lift” pack are likely to facilitate development of self-efficacy: they make it easy for the individual to close the container and thus reduce negative health outcomes (in this case, child poisoning risk). Self-efficacy is easily accomplished and bad habits (failing to close the container) are broken [10, 14].

Such strategies–making a healthy behaviour easy to accomplish and therefore improving underlying self-efficacy–have proven successful in many other domains of health-related behaviour. When schools provided free and readily available fruits and vegetables to families, for example, children’s fruit and vegetable intake increased [15]. In an example from commercial products, the addition of anti-lock brake systems to motorcycles resulted in a 37% decrease in fatal motorcycle crashes [16]. Introduction of packs like “Press & Lift” into the commercial market might have similar results. By making safe practices easy and accessible to perform, consumers are more likely to engage in them.

The concepts behind the “Press & Lift” pack design could be generalised to other uses. As an example, empirical evidence suggests many medication containers are difficult for consumers to open, especially older consumers and those with vision impairments [17, 18]. Creative engineering to identify packaging that is compliant with CIC regulations but is easier to open and close may lead to fewer unintentional child poisoning incidents from medications that result from containers left open or pills transferred to alternative storage locations that are easier to open and close.

If designs like “Press & Lift” were widely adopted, critics may be concerned that it would lead to the so-called “lulling effect”: users may assume their child is safe and risk is low, so complementary safety-promoting activities like supervision and childproofing decrease, sometimes called risk compensation or risk homeostasis [19, 20]. We believe that to be unlikely, as evidenced by parallel findings with safety modifications to products like disposable lighters [21] and use of bicycle helmets [22].

Like all research, our study had limitations. Study 1 relied only on self-report data, although that limitation was overcome through the objective sensor data collected in Study 2. Because child poisoning is a rare event, we relied on proxy measures to assess child poisoning risk. Large-scale epidemiological research might be conducted in the future to confirm our experimental findings. Both samples were relatively small, and data were collected in two cultures that have somewhat different laundry habits due to typical home construction but may not be representative of results in other cultures.

In conclusion, results from two studies indicate the “Press & Lift” pack may reduce chid poisoning risk because it is closed more consistently by users. It was rated as easier to open and to close, equally child safe, and a product that would simplify the laundry process by participants in both Germany and the United Kingdom. Objectively collected data replicated self-report findings of increased closure with the “Press & Lift” pack versus the previously-available “Pinch & Lift” system. Data indicate potential advantages of introducing systems like the “Press & Lift” system into the market to increase consumer satisfaction and reduce child poisoning risk.

## Supporting information

S1 File Data study 1. (XLSX)

S2 File Data study 2 claimed. (XLSX)

S3 File Data study 2 sensor. (XLSX)
